# The *Arabidopsis* MADS-Domain Transcription Factor SEEDSTICK Controls Seed Size via Direct Activation of *E2Fa*

**DOI:** 10.3390/plants10020192

**Published:** 2021-01-20

**Authors:** Dario Paolo, Lisa Rotasperti, Arp Schnittger, Simona Masiero, Lucia Colombo, Chiara Mizzotti

**Affiliations:** 1 Dipartimento di BioScienze, Università degli Studi di Milano, 20133 Milano, Italy; dario.paolo@ibba.cnr.it (D.P.); lisa.rotasperti@unimi.it (L.R.); simona.masiero@unimi.it (S.M.); lucia.colombo@unimi.it (L.C.); 2 Abteilung für Entwicklungsbiologie, Institut für Pflanzenforschung und Mikrobiologie, Universität Hamburg, 22609 Hamburg, Germany; arp.schnittger@uni-hamburg.de

**Keywords:** *Arabidopsis thaliana*, cell cycle, seed development, transcription factor

## Abstract

Seed size is the result of complex molecular networks controlling the development of the seed coat (of maternal origin) and the two fertilization products, the embryo and the endosperm. In this study we characterized the role of *Arabidopsis thaliana* MADS-domain transcription factor SEEDSTICK (STK) in seed size control. STK is known to regulate the differentiation of the seed coat as well as the structural and mechanical properties of cell walls in developing seeds. In particular, we further characterized *stk* mutant seeds. Genetic evidence (reciprocal crosses) of the inheritance of the small-seed phenotype, together with the provided analysis of cell division activity (flow cytometry), demonstrate that STK acts in the earlier phases of seed development as a maternal activator of growth. Moreover, we describe a molecular mechanism underlying this activity by reporting how STK positively regulates cell cycle progression via directly activating the expression of *E2Fa*, a key regulator of the cell cycle. Altogether, our results unveil a new genetic network active in the maternal control of seed size in *Arabidopsis*.

## 1. Introduction

In spermatophyte plants, reproductive success depends on the ability to produce healthy seeds. Indeed, seed size represents one of the major traits that influence the fitness of the next plant generation. Despite their importance, molecular regulators of seed size have only begun to be identified in the last few years, mainly through studies on *Arabidopsis* [1,2,3,4]. Critical factors governing seed size include the parent-of-origin effect in fertilization tissues, overall plant fertility (balance between seed size/number), cell cycle/cell expansion regulation and hormonal signaling [5,6,7,8].

The final size of *Arabidopsis* seeds is achieved through coordinated growth of the three parts that compose the seeds: the seed coat, the endosperm and the embryo [9]. All three parts are characterized by a different genotype: the seed coat, deriving from the ovule integuments, is entirely of maternal origin, whereas both embryo and endosperm are the result of double fertilization. In *Arabidopsis*, seed size is established around four days after fertilization (DAF), when the dimension of the seed cavity has reached its maximum expansion as a result of seed coat growth and endosperm proliferation [10,11].

Many factors influencing ovule and seed development have been identified [1,12]. In particular, MADS-domain transcription factors have been found to be key determinants of female reproductive development [13]. Among them, the *Arabidopsis* transcription factor SEEDSTICK (STK) has been largely studied in relation to its function in ovule identity determination [14,15], transmitting tract development [16,17] seed abscission [15,18], seed coat development [19] and flavonoid biosynthesis [20]. We have also previously shown how STK regulates size and differentiation of fruits by providing a link between signaling hormones (cytokinins) and other molecular developmental networks [21]. Loss-of-function *stk* mutants produce smaller seeds than wild-type, albeit with no obvious reduction in overall fertility [15]. However, the underlying molecular mechanisms of how STK promotes seed growth are not very well understood. We therefore conducted additional studies on the genetics of the small-seed phenotype of the *stk* mutant and we analyzed differences in mitotic activities emerging from the comparison with wild-type seeds. Moreover, we investigated possible downstream targets of STK involved in cell cycle progression. In this frame, we focused on E2Fa, a crucial transcription factor that regulates the mitotic activity of cells that is also co-expressed with STK in the seed coat of developing seeds [22,23,24]. The single *e2fa* mutant does not have a seed-related phenotype, while the double mutant *e2fa*/*b* has enlarged seeds, an indirect effect probably due to reduced fertility of the mutant which therefore diverts more allocated resources to the surviving seeds [24]. Here, we provide evidence of the involvement of STK in cell cycle control via the positive regulation of the expression of *E2Fa* in developing seeds.

## 2. Results and Discussion

### 2.1. STK Maternally Controls Seed Size and Controls Cell Cycle Progression in Seeds

**Figure 1:**
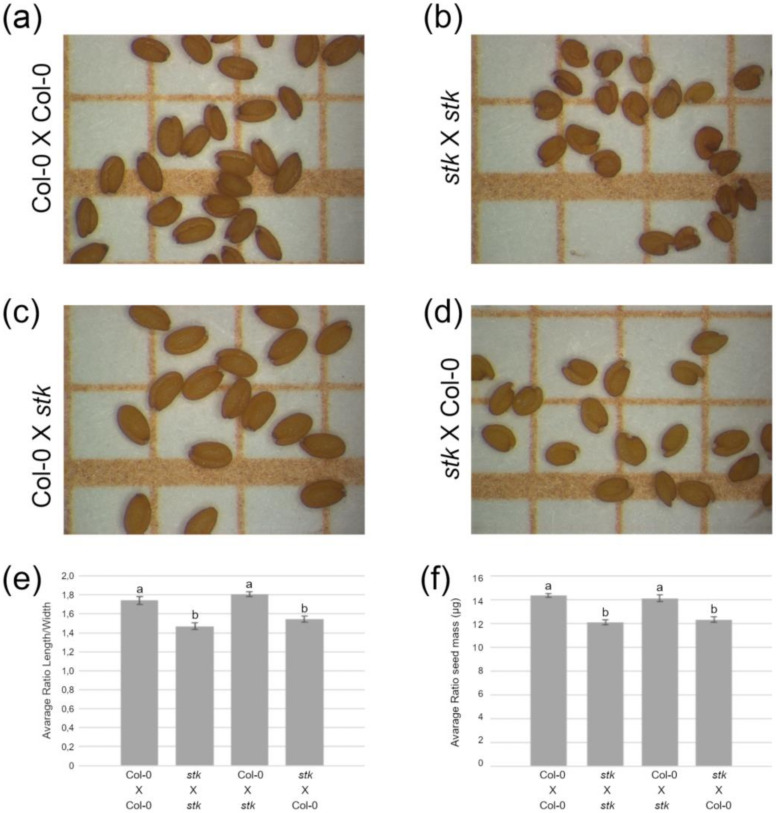
The phenotype of *stk* mutant seeds has a maternal sporophytic origin. (**a**–**d**) Seeds of reciprocal crosses between *stk* and wild-type Columbia (Col-0) plants on 1 mm squared graph paper. (**e**) Ratio between seed length and width. (**f**) Seed mass. Bars indicate means plus standard error; different letters above bars indicate statistically significant differences (*p* < 0.01).

To further characterize the reduction in seed size of *stk* seeds, we performed reciprocal crosses with wild-type plants. Our data confirmed that the smaller size and the rounder shape of *stk* seeds (Figure 1b,d,e) in comparison to wild-type (Figure 1a,c,e) only depend on the maternal origin of the seed coat, as suggested previously [15,25]. The maternally inherited small-seed phenotype of *stk* is also reflected in the reduction of average seed mass (Figure 1f).

A key parameter of organ growth is cell number, which is determined by cell division activity. Determining the ratio between 4C and 2C cells, i.e., before and after mitosis, can give a first estimate of cell cycle activity. In young seeds (0–6 DAF), the 4C/2C ratio is mainly influenced by seed coat cells, which are the predominant fraction of cells at this time point [26].

Previous flow cytometry analyses revealed that wild-type and *stk* seeds differ in terms of 4C and 2C peaks [25]. In *stk* seeds, nearly 50% of the cells reside in the gap before mitosis, whereas this is only the case for 35% of the cells in wild-type seeds (Supplementary Figure S1). This suggests that *stk* mutants either progress faster through the S-phase or are slowed down in their progression through mitosis.

**Figure 2:**
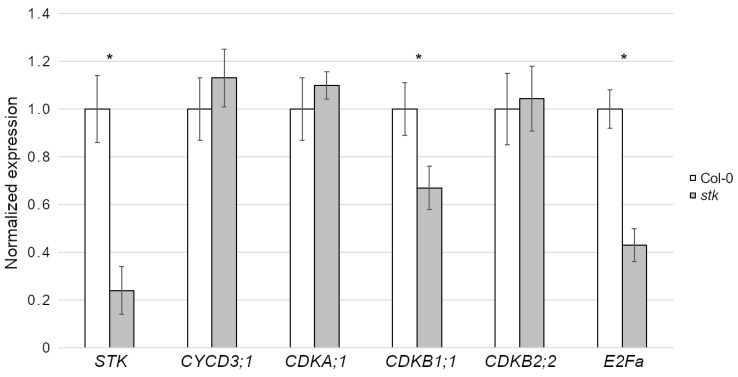
Expression level of cell cycle regulators in Col-0 and *stk* mutants. The q-PCR was conducted in triplicates using *ACTIN* (*ACT*) and *UBIQUITIN* (*UBQ*) as internal reference genes (Supplementary Table S1). Error bars represent the propagated standard deviation error value using three replicates; asterisks represent statistically significant differences (*p* < 0.01).

Next, we investigated how *STK* could control cell proliferation by q-PCR (Figure 2), monitoring the expression levels of known cell cycle regulators, including transcription factors, cyclins (CYC) and cyclin-dependent kinases (CDK).

In *Arabidopsis*, the combined kinase activities of *CYCD3;1* and *CDKA;1* constitute a major positive force of cell cycle progression [27,28,29]. Despite evidence of an altered cell cycle progression in the seed coat of *stk* seeds, as shown by the flow cytometry analysis, we did not report significant downregulation of *CYCD3;1* nor *CDKA;1* (Figure 2). The checkpoint between the G2 and M phases is also controlled by CDKs of Type B. In particular, B1-type CDKs are characterized by maximum kinase activity during the transition from G2 to M phase, while B2-type CDKs reach their maximum activity during mitosis [30]. However, *CDKB1;1* is only slightly downregulated in *stk* and *CDKB2;2* expression is not affected in the mutant (Figure 2).

In proliferating cells, the transcription factor E2Fa acts in complex with RBR1 (RETINOBLASTOMA-RELATED PROTEIN 1) to maintain proliferation competence by inhibiting genes that control the switch from mitosis to the endocycle [31]. Moreover, similar to the STK protein, E2Fa accumulates in the nucleus of the outer integument cells [24]. For this reason, we tested the expression of *E2Fa* and interestingly, our analysis demonstrated that developing *stk* mutant seeds showed a partially reduced *E2Fa* expression in comparison to wild-type ones (Figure 2).

Since E2Fa-induced genes also encode proteins involved in cell wall biosynthesis, providing a possible link to STK function [19,32,33], we further investigated a possible regulation of *E2Fa* via STK.

### 2.2. STK Directly Regulates E2Fa

**Figure 3:**
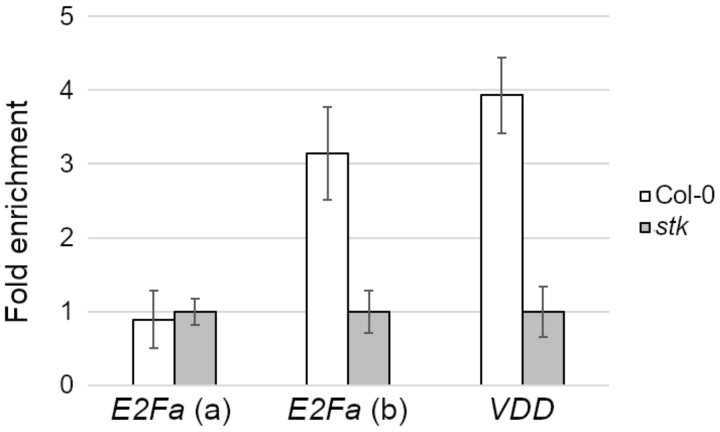
Chromatin immunoprecipitation (ChIP) enrichment by q-PCR of the STK binding site on *E2Fa*. Region “b” of *E2Fa* (see Supplementary Figure S2) is directly bound by STK during seed development, while region “a” is not. *VERDANDI* (*VDD*) was used as positive control. The q-PCR was conducted in duplicates. Fold enrichment was calculated over the negative controls. Error bars represent the propagated error value using three replicates.

To understand the nature of *E2Fa* regulation via STK, we screened the *E2Fa* gene sequence and found two putative CaRG boxes, the CC[A/T]_6_GG consensus sequences known to be recognized by the MADS-domain protein [34,35] (Supplementary Figure S2). To test if *E2Fa* could be a direct target of STK, we performed a chromatin immunoprecipitation (ChIP) assay using an anti-STK antibody (Figure 3). As a positive control, we tested *VERDANDI* (*VDD*), a known direct target of STK [35]. Immunoprecipitated chromatin obtained from 0–4 DAF fruits was tested via q-PCR analysis, which showed that the putative binding site “b” of STK on *E2Fa* was significantly enriched over the negative control (Figure 3). This data indicates that *E2Fa* expression is directly regulated by STK in developing seeds by the binding to regulative region “b”.

**Figure 4:**
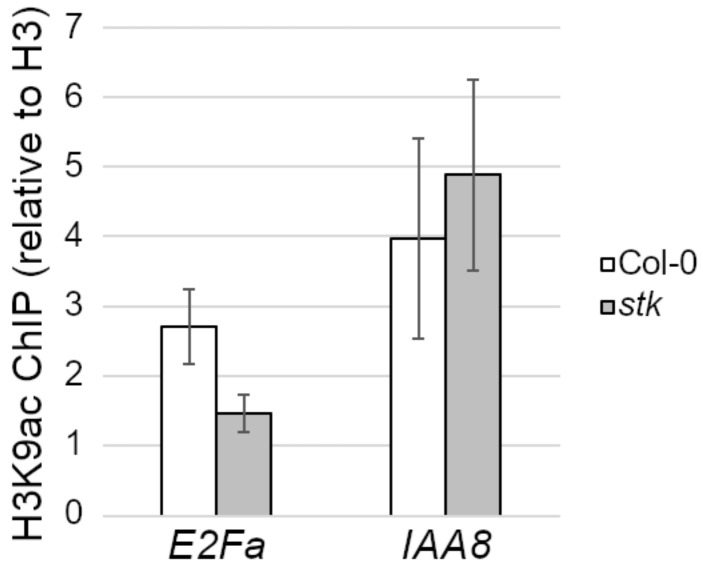
ChIP enrichment tests by q-PCR of H3-lysine9 acetylation (H3K9ac) level in *E2Fa* region “b” of wild-type and *stk* samples expressed as % input, normalized on total H3 level with error bars representing the propagated error value using three replicates. *IAA8* was used as positive control.

Previous studies indicate that protein complexes involved in chromatin remodeling can be recruited on target genes by transcription factors of different families, including MADS [36]. For this reason, we investigated the chromatin landscape at the confirmed STK binding site on *E2Fa*, as we had previously done for *BANYULS (BAN),* another direct target of STK during the development of the seed coat [20]. This was done by means of a ChIP experiment on both wild-type and *stk* 0–4 DAF fruits, using a specific antibody to detect the level of H3-lysine9 acetylation (H3K9ac), an epigenetic mark usually associated with the activation of expression, choosing *INDOLE ACETIC ACID-INDUCED PROTEIN 8* (*IAA8*) as a positive control [37]. Indeed, we revealed a lower level of H3K9ac in the *stk* mutant background versus the wild-type (Figure 4), in accordance with our hypothesis of a direct positive regulation of *E2Fa* expression levels by STK.

## 3. Conclusions

Based on our previous analyses [20,25] and the data presented here, we propose that STK controls cell cycle progression in the developing seed coat. 

This mechanism involves the positive regulation of *E2Fa*. We also found that *CDKB1;1* expression is reduced and interestingly, the promoter of *CDKB1;1* is bound and regulated by E2Fa [38]. The activation of *E2Fa* likely involves local epigenetic remodeling of chromatin, as seen by the reduction in open and active chromatin at the STK-bound *E2Fa* locus in *stk* mutants. Thus, the regulation of ovule and seed development by STK expands to cell proliferation control, opening a new facet of MADS–domain transcription factors’ action during reproduction.

## 4. Materials and Methods

### 4.1. Plant Material and Growth Condition

*Arabidopsis thaliana* wild-type (ecotype Columbia, Col-0) and *stk* mutant lines were grown on soil at 22 °C (3–6 plants per pot), initially in growth chambers under short-day conditions (8 h light/16 h dark) for two weeks and then moved to an experimental greenhouse under long-day conditions (16 h light/8 h dark). *stk* mutant lines are homozygous for the *stk-2* allele, which contains a 74 nucleotide insertion near the splice site of the 3rd intron; *stk* seeds were obtained by M. Yanosfky’s lab [15].

### 4.2. Genotyping

PCR-based genotyping for mutant alleles was performed with the primers listed in Supplementary Table S1 with the following conditions: 95 °C 3′, 35 cycles of 95 °C 30″ + 61 °C 30″ + 72 °C 30″, 72 °C 5′ and PCR products were checked on ethidium bromide-stained 1% agarose gel in 1 × TAE (Tris base, acetic acid and EDTA buffer).

### 4.3. Seed Analysis

Seeds were photographed using a Leica MZ6 stereomicroscope, and seed images (*n* = 25) were measured using ImageJ software. Average seed mass was determined by weighing mature dry seeds in batches of 500 and the weights of at least three replicates were measured for each seed lot. Measurements were statistically analyzed by one-way ANOVA with post-hoc Tukey honestly significant difference (HSD) comparison test and statistical differences marked as different letters reflect *p* < 0.01.

### 4.4. Ploidy Analysis

For flow cytometry analysis, flowers were emasculated 24 h before manual pollination with pollen of the same genotype. Seeds were collected from the siliques at 6 DAF and prepared for analysis using the CyStain^®^ UV Precise kit (Partec, Cat. 05-5002). The staining with DAPI (4′,6-diamidino-2-phenylindole) was performed as previously described [39]. The ploidy level was calibrated against the 2C nuclear DNA content peak derived from a preparation of young rosette leaves [25].

### 4.5. Expression Analyses

Total RNA was extracted in triplicate from flowers and siliques until 4 DAF using the LiCl method [40]. Total RNA was treated using Ambion TURBO DNA-free^TM^ DNase (Ambion, Cat. AM1907) and then reverse transcribed using the Bio-Rad iScript^TM^ kit (Bio-Rad, Cat. 170-8891). q-PCR was performed on three biological samples and on technical triplicates using the iQ5 real-time PCR detection system (Bio-Rad) in 20 µL reactions with 250 nM of each primer and the SYBR Green PCR Master Mix (Bio-Rad). The 3-step amplification protocol used was 95 °C 3′ followed 40 cycles of 95 °C 10″ + 60 °C 30″, with melt curves calculated over the 60–95 °C interval with +0.5 °C increments. cDNA was standardized relative to *UBIQUITIN* (*UBQ*) and *ACTIN* (*ACT*) transcript levels and gene expression analysis was performed with the 2^−ΔΔCt method using Bio-Rad CFX Maestro software v.4.0 (Bio-Rad). Baseline and threshold levels were set according to the manufacturer’s instructions. Student’s *t*-tests were run for each experiment and asterisks indicate significant differences (*p* < 0.01). The primers used in these experiments are listed in Supplementary Table S1.

### 4.6. Chromatin Immunoprecipitation Assay

ChIP experiments to evaluate the enrichment of *E2Fa* were performed with an antibody specific for the STK protein on fertilized flowers and siliques until 4 DAF. Plant material was collected from wild-type (Col-0) and *stk* plants. The q-PCR assay was conducted on biological duplicates, with three technical replicates for each sample. The q-PCR protocols used were the same as those listed in Section 4.5 of this manuscript. Data were obtained using the iQ5 real-time PCR detection system (Bio-Rad) with the SYBR Green PCR Master Mix (Bio-Rad) and, for the region of interest, fold enrichment over negative control (*ACT*) was evaluated with a method previously reported using *VDD* as a positive control [35].

Similarly, relative differences in acetylation levels between the wild-type (Col-0) and the *stk* mutant were tested by ChIP using the same developmental stages mentioned above. We used an antibody against an unmodified isoform of H3 (“total H3”, Upstate, Cat. 06-753) and one specific for H3K9ac (Upstate, Cat. 07-532). For each sample, the percentage of enrichment in the acetylation versus input was calculated as previously shown [41], normalizing for total H3. *IAA8* was used as a positive control [20]. The primers used in these experiments are listed in Supplementary Table S1.

## Data Availability

The data presented in this study are available on request from the corresponding author.

## Supplementary Materials

The following are available online at https://www.mdpi.com/2223-7747/10/2/192/s1, Figure S1: DNA-content profile obtained from flow cytometry analysis of seeds; Figure S2: Schematic representation of the *E2Fa* gene; Table S1: List of primers.
