# Supplementary material for: The Arabidopsis MADS-Domain Transcription Factor SEEDSTICK Controls Seed Size via Direct Activation of E2Fa
Source: Plants (Basel). 2021 Jan 20;10(2):192. doi: 10.3390/plants10020192 (PMC7909557; doi:10.3390/plants10020192)
Supplement: Supplementary file 1 [file plants-10-00192-s001.pdf]

plants

MDPI

Communication

The Arabidopsis MADS-domain transcription factor SEED-STICK controls seed size via direct activation of *E2Fa*.

Dario Paolo [1,†], Lisa Rotasperti [1], Arp Schnittger [2], Simona Masiero [1], Lucia Colombo [1] and Chiara Mizzotti [1,*]

1 Dipartimento di BioScienze, Università degli Studi di Milano, 20133 Milano, Italy; lisa.rotasperti@unimi.it (L.R.); simona.masiero@unimi.it (S.M.); lucia.colombo@unimi.it (L.C.)
2 Abteilung für Entwicklungsbiologie, Institut für Pflanzenforschung und Mikrobiologie, Universität Hamburg, 22609 Hamburg, Germany; arp.schnittger@uni-hamburg.de
* Correspondence: chiara.mizzotti@unimi.it; Tel.: +39-02-503-14838
† Present address: Institute of Agricultural Biology and Biotechnology, CNR, 20133 Milan, Italy; dario.paolo@ibba.cnr.it

Citation: Paolo, D.; Rotasperti, L.; Schnittger, A.; Masiero, S.; Colombo, L.; Mizzotti, C. The Arabidopsis MADS-Domain Transcription Factor SEEDSTICK Controls Seed Size via Direct Activation of *E2Fa*. *Plants* **2021**, *10*, 192. https://doi.org/10.3390/plants10020192

Received: 23 December 2020
Accepted: 16 January 2021
Published: 20 January 2021

Publisher's Note: MDPI stays neutral with regard to jurisdictional claims in published maps and institutional affiliations.

Copyright: © 2021 by the authors. Licensee MDPI, Basel, Switzerland. This article is an open access article distributed under the terms and conditions of the Creative Commons Attribution (CC BY) license (http://creativecommons.org/licenses/by/4.0/).

Supplementary Figures

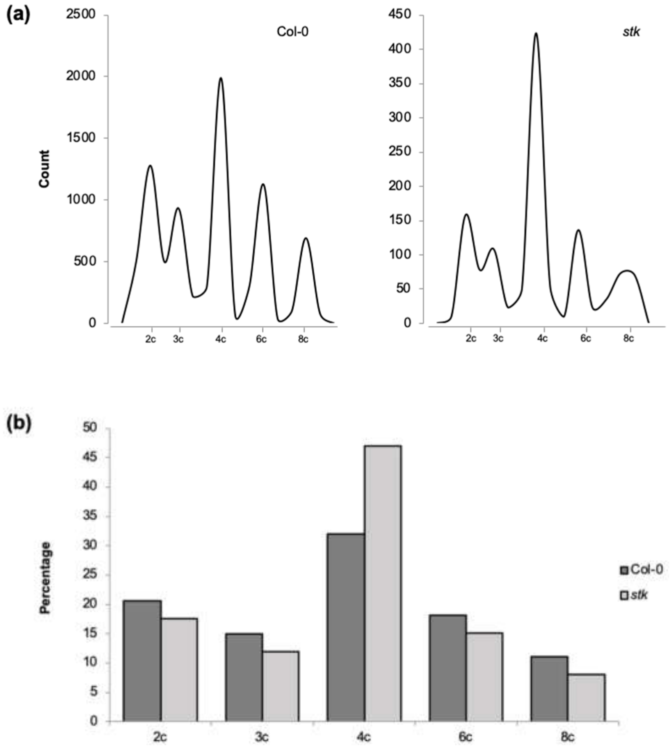

Supplementary Figure S1. DNA-content profile obtained from flow cytometry analysis of seeds. (**a**) DNA-content profile of wild-type (Col-0) and of *stk* mutant seeds showing the different peaks related to diploid nuclei (2C, 4C), highly endo-reduplicated nuclei (8C) and triploid/endospermal nuclei (3C, 6C). (**b**) quantitative representation of each peak relative to total measured samples.

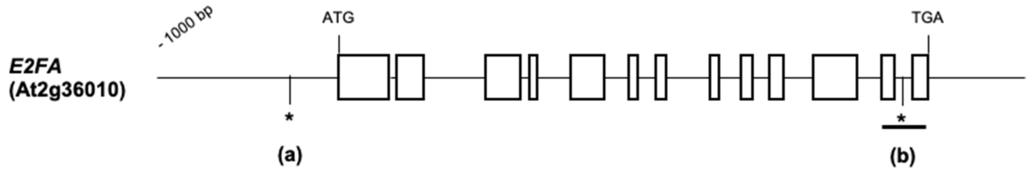

Supplementary Figure S2. Schematic representation of *E2Fa* gene with intron/exon structure, with asterisks indicating the positions of CArG boxes at the putative "a" and "b" binding sites of STK.

Supplementary Table 1. Primers used in this work

**Primers for genotyping**	
stk-2	5′-GCTTGTTCTGATAGCACCAACACTAGCA-3′
	5′-CCACTAACCATTTGATGATGGTGTTGT-3′
**Primers for expression analysis**	
STK	5′-ACGCGCAGAAAAGGGAGATTGAGC -3′
At4g09960	5′- TGTCGGGATCAGAGTAAGAACCTCC -3′
CYCD3;1	5′- CCAAACTAATCTCCTCGGTGTCC -3′
At4g34160	5′- TGATGATGAATCGTGACTCTTGCG -3′
CDKA;1	5′- CTGACAGGGATACCGAATGCTC -3′
At3g48750	5′- GGAGAAGGAACATACGGGAAAGTC -3′
CDKB1;1	5′- GGAGAAGGAACATACGGGAAAGTC -3′
At1g76540	5′ ACGAAGAGCAGTTGGTGGAATAC -3′
CDKB2;2	5′- AGAGATTGATAGAGATGGACAACAAT -3′
At1g20930	5′- AGGATCACGAGCGAGCATAC -3′
E2Fa	5′- CTTTGGAAGGGAGTTGATGCGTGTC-3′
At2g36010	5′- TTGTGCCATGAGGAGCTTTGACG -3′
ACT	5′- CTCAGGTATTGCAGACCGTATGAG -3′
At3g18780	5′- CTGGACCTGCTTCATCATACTCTG -3′
UBQ	5′- CTGTTCACGGAACCCAATTC -3′
At4g05320	5′- GGAAAAAGGTCTGACCGACA -3′
**Primers for ChIP experiment**	

E2Fa – region a	5′- AGCGGTACGAAAACTTTGCC -3′
	5′- GTTGTATCATGTATGGCAGTGG -3′
E2Fa – region b	5′- ACTGGCTTCTCTCAAATGCTGAG -3′
	5′- GGTGCTATTTCGCCCATTCCT -3′
VDD	5′- GGAAATATGACGCTTGTCTTTTTAG -3′
At5g18000	5′- CAGAAACAGCAATATGCTCGTG -3′
IAA8	5′- CAATGGCTTCTTCTACTTCG -3′
At2g22670	5′- CCAATCACCGTCTTTATCT -3′
ACT	5′- CGTTTCGCTTTCCTTAGTGTTAGCT -3′
At3g18780	5′- AGCGAACGGATCTAGAGACTCACCTTG -3′
